# The rocking line illusion

**DOI:** 10.1177/20416695231184388

**Published:** 2023-07-04

**Authors:** Ian M. Thornton, Dejan Todorović

**Affiliations:** Department of Cognitive Science, Faculty of Media and Knowledge Sciences, University of Malta, Msida, Malta; Laboratory of Experimental Psychology, Department of Psychology, University of Belgrade, Belgrade, Serbia

**Keywords:** visual illusions, motion, orientation

## Abstract

A new visual illusion is described in which a smoothly translating object appears to rock around its own center during motion. This “rocking line” illusion occurs when the object passes through contrast boundaries formed by static background elements. However, for it to appear, the spatial scale of the display must be adjusted appropriately. We provide an online demo where the effect can be experienced and relevant parameters manipulated.

It is well known that the contrast between the background and inducing elements in static displays can give rise to strong orientation illusions. The café wall illusion (Gregory & Heard, 1979) and Akiyoshi Kitaoka's compelling effects (e.g., Kitaoka, 2007) are probably the best-known examples (see Todorović, 2021 for a review). Here, we describe a novel dynamic illusion that may rely on similar mechanisms. The “rocking line” illusion (RLI) involves a rectangular target object (Figure 1a), and a background context consisting of a series of horizontally staggered rectangles that span the screen midline, forming a narrow checkboard pattern with the same dimensions as the target (Figure 1b). When the target smoothly translates along the midline of the checkerboard, two very different percepts occur, depending crucially on spatial scale/viewing distance.

**Figure 1.**
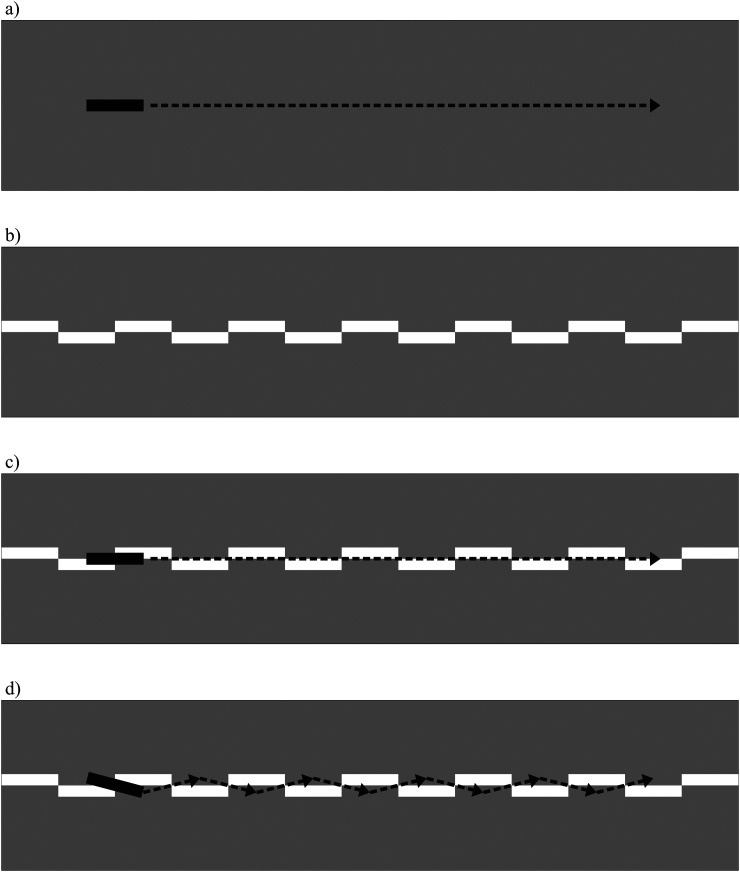
Components and phenomenology of the “rocking line” illusion (RLI). See the text for details.

With relatively large elements (all rectangles ≃2° VA), a veridical impression of horizontal motion occurs (Figure 1c). However, when the display is scaled down in size—or the observer simply moves back slightly—a compelling impression that the rectangle rocks around its own center begins to dominate (Figure 1d). While Figure 1 provides a rough illustration of the RLI, these effects can be more directly experienced online at https://maltacogsci.org/RLI/. See also the OSF page associated with this work at https://osf.io/sqfex/.

The RLI appears to be robust, with all observers reporting a clear impression of rocking at some spatial scale. In informal demonstrations and at scientific meetings, those sitting furthest from the screen are first to report a deviation from horizontal motion. To more formally document the effect, we presented the display to 10 naïve observers under laboratory conditions. They were seated 57 cm in front of the display with their head position fixed via a chin rest, so that the background checkboard extended for 20°×0.8° and each display rectangle subtended 2°×0.4°. The observers were asked to report when the motion of the rectangle appeared to deviate from horizontal and were explicitly told to expect some form of “rocking” of the trajectory. A response—rocking or horizontal—was taken at each of the 10 scales, with the rectangle sizes varying in serial order from 2° down to 0.2°, and then back up in the same steps to 2°. Results are summarized in Figure 2. It is clear that the tendency to see the illusion increases as size decreases, with this pattern reversing as the target again becomes larger. We note that while all observers reported normal or corrected to normal vision, at the smallest display size (0.2°), several reported being unable to resolve the target object, providing a possible explanation for the dip of responses at this scale.

**Figure 2.**
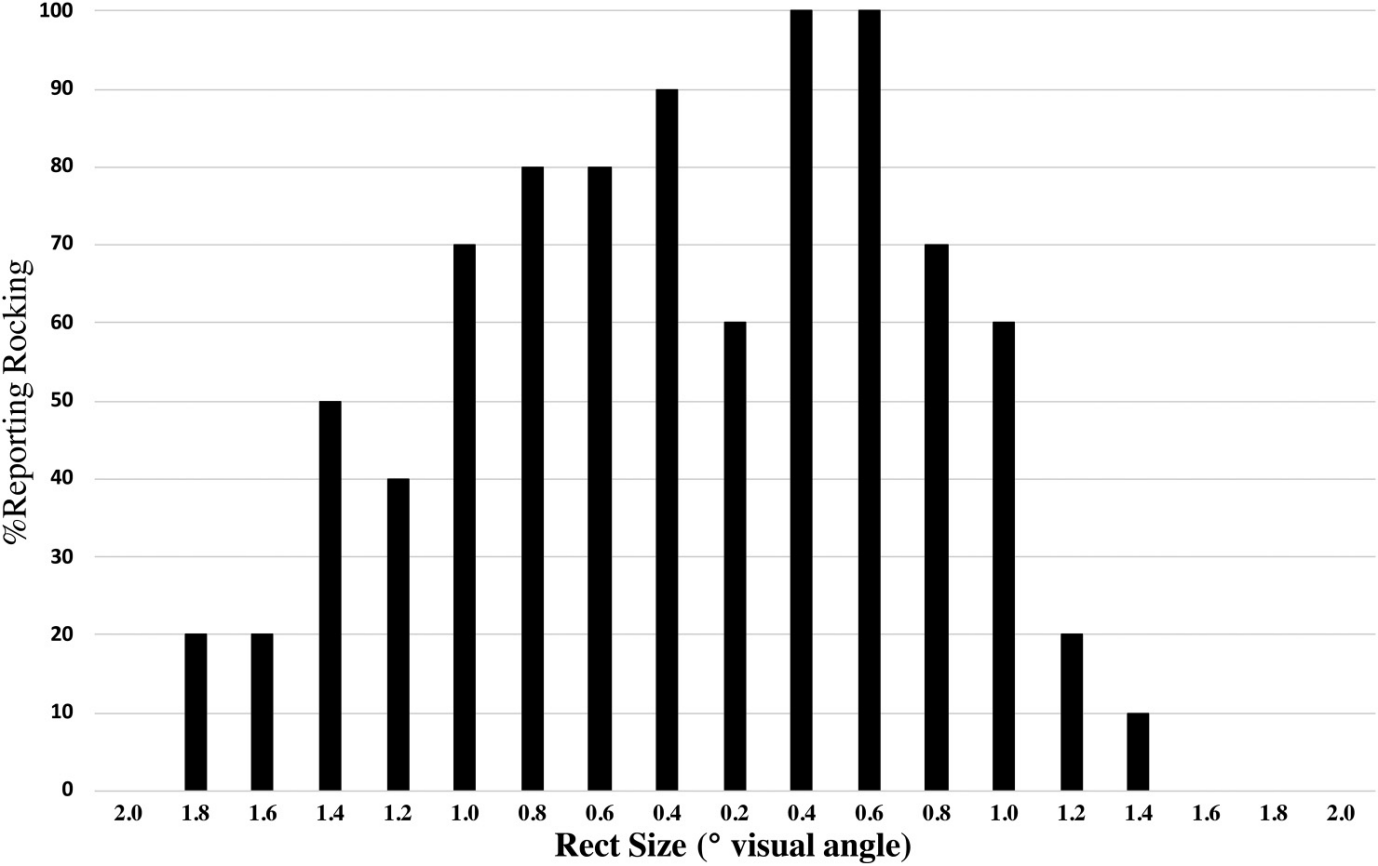
Percentage of observers reporting the “rocking line” illusion (RLI) as a function of target size, which was decreased, then increased in serial order during the experimental presentation to simulate the effects of scaling in the online demo.

Some additional observations—which can be verified using the online demo—are as follows. The RLI does not require strong contrast boundaries. Indeed, the effect seems somewhat strengthened when the background elements are faded, and remains intact even if the background and target element have the same polarity (i.e., are both increments or decrements relative to the surround).

The RLI is also not directly dependent on the speed of motion. While increasing speed may enhance the effect, a shift in orientation can also be experienced with extremely slow motion. Indeed, a reduced illusory tilt may even be experienced when motion is stopped, particularly when the number of target objects is increased (see demo “Multi RLI” option). As described below, this suggests a common causal mechanism with static orientation illusions (Kitaoka, 2007; Todorović, 2021). Motion thus appears to amplify the sense of illusory tilt, while additional dynamic grouping mechanisms (e.g., common fate) probably explain why the whole object appears to “rock,” rather than the leading edge appearing to break away from the portion that is still relatively far from the junction. Our informal observations with elongated moving objects further support the influence of spatial integration. That is, when the length of the moving object is increased, the entire object appears to nonrigidly deform—a rocking snake illusion?—rather than separating into horizontal and tilted segments. At slow speeds, the influence of the horizontal contrast borders away from the checkboard boundaries also becomes more obvious, suggesting a link to the footstep/inchworm illusion (Anstis, 2001; Kitaoka & Anstis, 2021). We are also exploring possible connections to the slalom illusion (Cesàro & Agostini, 1998).

Having described the basic RLI, and some of the factors that appear to influence it, two important questions emerge. First, what causes the illusory shift in orientation? Second, why is the effect so dependent on spatial scale?

Todorović (2021), in his account of *static* “polarity-dependent orientation illusions,” used simulations to show that an impression of tilt may arise due to “oblique clusters” of neural activity emerging from the output of two types of horizontally tuned simple cells when an object straddles a contrast border. This idea is sketched in Figure 3 (see Todorović, 2021 for model details). With the RLI, such oblique clusters would be expected to appear each time the object moves across an X-junction in the background checkerboard. Initial simulations of the pattern of activity arising during object motion do indeed show the transient appearance of such oblique clusters. These can be viewed at https://maltacogsci.org/RLI/simulation.gif, and on the OSF page associated with this work at https://osf.io/sqfex/.

**Figure 3.**
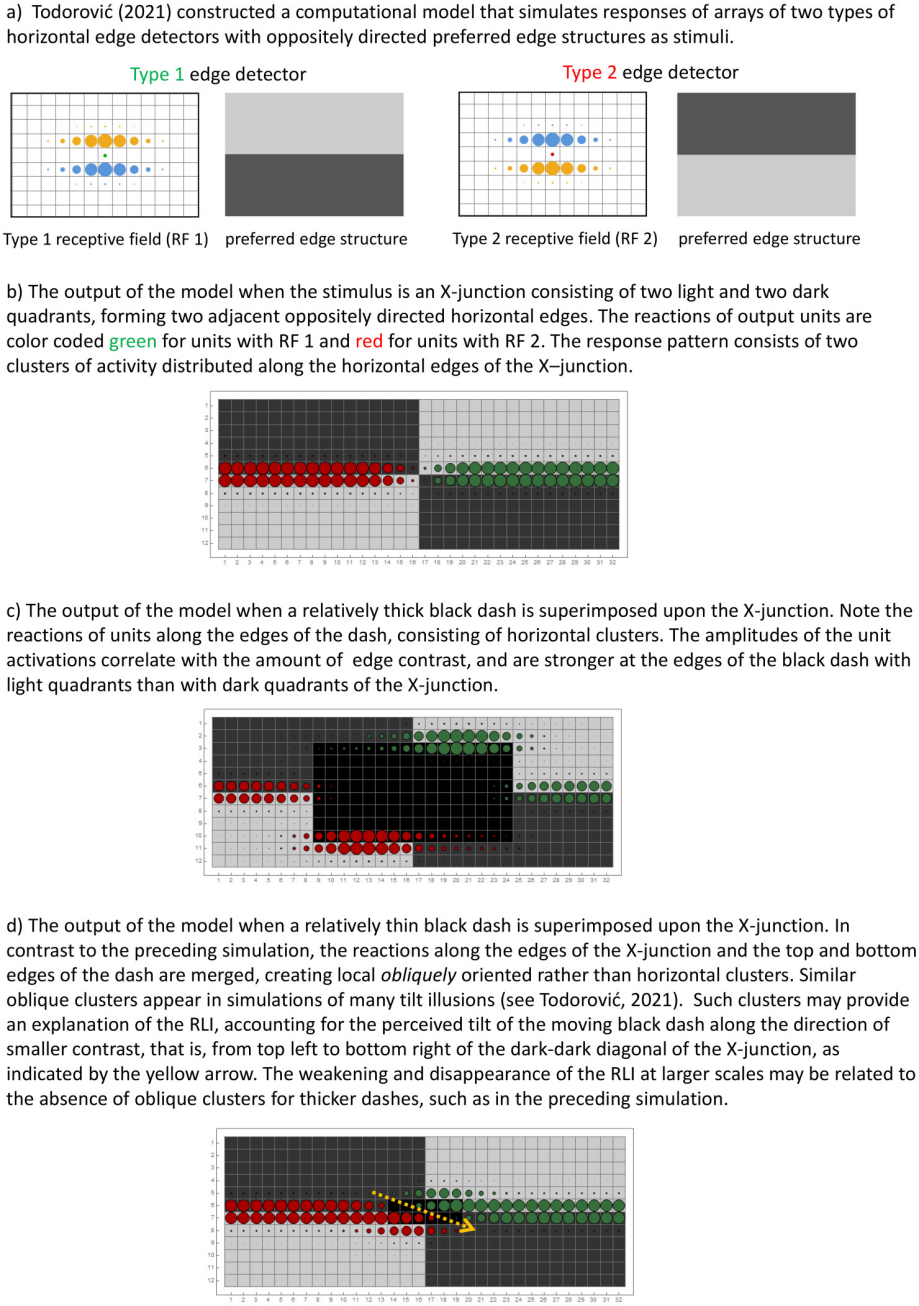
Simulation receptive field structure and output. See Todorović (2021) for details.

Finally, why does the RLI depend on the spatial scale? Our current suggestion, based on initial simulations, is that this is due to the fact that scale affects the distance between the checkerboard midline and the top and bottom target edges (see Figure 3c–d). At larger scales, all these horizontal edges are associated with corresponding separate “horizontal” simulated neural activity clusters. As the scale decreases, these clusters begin to merge and form emergent “oblique” clusters, whose orientation is in accord with the phenomenology of the illusion.
